# TUBB2A and TM4SF1 Methylation Define Prognostic Subgroups in Adult-Type Diffuse Glioma

**DOI:** 10.3390/cancers18040638

**Published:** 2026-02-16

**Authors:** Tarek Arabi, Itika Arora, Arshiya Akbar, Naveed Syed Abdul Kareem, Zara Ahmed, Volodymyr Mavrych, Olena Bolgova, Shamsa Hilal Saleh, Firoz Ahmed, Ahmed Abu-Zaid, Edwin N. Aroke, Mohammed Imran Khan, Ahmed Yaqinuddin

**Affiliations:** 1 College of Medicine, Alfaisal University, Riyadh 11533, Saudi Arabia; tarabi@alfaisal.edu (T.A.); itika.arora@ku.ac.ae (I.A.); arshiyaakbar2019@gmail.com (A.A.); nabdulkareem@alfaisal.edu (N.S.A.K.); zaahmed@alfaisal.edu (Z.A.); vmavrych@alfaisal.edu (V.M.); obolgova@alfaisal.edu (O.B.);; 2 Center for Biotechnology, Khalifa University of Science and Technology, Abu Dhabi 127788, United Arab Emirates; 3 King Faisal Specialist Hospital and Research Centre, Riyadh 111211, Saudi Arabia; 4 Department of Biological Sciences, University of Jeddah, Jeddah 23218, Saudi Arabia; fahmed@uj.edu.sa; 5 Department of Acute, Chronic & Continuing Care, School of Nursing, The University of Alabama at Birmingham, Birmingham, AL 35294, USA; earoke@uab.edu; 6 King Faisal Specialist Hospital and Research Centre, Jeddah 21499, Saudi Arabia

**Keywords:** glioblastoma, DNA methylation, glioma-CIMP (G-CIMP), prognostic biomarkers, gene-specific methylation

## Abstract

**Background/Objectives:** Gliomas arise from distinct subgroups driven by different genetic alterations. Gain-of-function mutations in isocitrate dehydrogenase 1/2 (IDH1/2) are frequent in a subset of gliomas and are associated with improved survival compared with wild-type tumors. IDH-mutant gliomas can be classified as having the CpG island methylator phenotype (G-CIMP), which is characterized by widespread epigenetic changes associated with IDH1 mutations. G-CIMP-based stratification provides an added improvement in glioma classification when considered alongside grade and histology. Despite the success of G-CIMP-based stratification in predicting outcome in some patients, it does not fully account for non-responders, largely due to heterogeneity within G-CIMP gliomas. **Methods:** Using a The Cancer Genome Atlas (TCGA) glioblastoma, IDH-wildtype discovery cohort, we identified gene-level DNA methylation and expression patterns associated with clinical outcomes, including epigenetically regulated genes such as *TUBB2A* and *TM4SF1*. **Results:** In an independent TCGA diffuse glioma validation cohort enriched for IDH-mutant tumors, canonical IDH-defined G-CIMP classification was applied, and promoter methylation of *TUBB2A* and *TM4SF1* showed significant associations with overall survival. **Conclusions:** These findings highlight molecular heterogeneity within IDH-defined G-CIMP gliomas and demonstrate that gene-specific methylation events can refine prognostic stratification beyond IDH status alone.

## Simple Summary

Adult-type diffuse gliomas are brain tumors that often look similar under the microscope but behave very differently in patients. Current markers, such as IDH mutation and MGMT promoter methylation, do not fully explain why some patients live much longer than others. In this study, we analyzed DNA methylation and gene expression data from two large The Cancer Genome Atlas cohorts of glioblastoma and lower-grade gliomas. We focused on two genes, *TUBB2A* and *TM4SF1*, whose activity can be switched off by methylation in their promoter regions. We found that higher promoter methylation of these genes, which corresponds to lower gene expression, was consistently linked to better overall survival, even after accounting for age, tumor grade, IDH status, and 1p/19q codeletion. Our results show that *TUBB2A* and *TM4SF1* methylation can define clinically meaningful prognostic subgroups and may help refine risk stratification and treatment planning for patients with adult-type diffuse glioma.

## 1. Introduction

Glioblastoma (GBM) is an aggressive brain tumor with inferior outcomes, as median survival remains roughly 15 months despite intensive therapy [1]. Although genetic changes play a role [2], significant epigenetic abnormalities also contribute to GBM’s aggressive behavior [3,4]. One of the most notable epigenetic features is abnormal DNA methylation. Promoter CpG island hypermethylation can transcriptionally silence key tumor-suppressor genes (e.g., *RB1*, *TP53*, *PTEN*) and pathways, whereas global hypomethylation of intergenic regions may activate oncogenes and promote genomic instability [5,6]. Such DNA methylation changes have direct clinical relevance. For example, epigenetic silencing of the O6-methylguanine DNA methyltransferase (*MGMT*) gene via promoter hypermethylation impairs DNA repair and sensitizes GBMs to temozolomide, correlating with significantly improved patient survival [7,8,9].

In this context, a distinct hypermethylation pattern, the CpG island methylator phenotype (CIMP), has attracted considerable interest. CIMP refers to coordinated hypermethylation of many gene promoters, first noted in colorectal cancer [10] and later identified in many other malignancies [11], including gliomas [12]. In diffuse gliomas, glioma-CIMP (G-CIMP) is strongly associated with IDH1/2 mutations and defines a distinct epigenetic subclass [12,13]. Nearly all IDH-mutant lower-grade gliomas exhibit a G-CIMP profile, and these G-CIMP-positive tumors are associated with substantially better outcomes than their CIMP-negative counterparts [12,13,14]. IDH mutations establish the G-CIMP phenotype through production of 2-hydroxyglutarate, which inhibits TET-mediated DNA demethylation and leads to widespread hypermethylation [15]. By contrast, CIMP is comparatively rare in IDH-wild-type GBM [16], which typically displays only limited or focal promoter methylation changes.

Recent studies indicate that certain IDH–wild-type GBMs can exhibit a CIMP-like hypermethylation pattern. Notably, methylation profiling has revealed an IDH-independent CIMP-like subset within *RTK2*-subtype GBMs [17]. These findings suggest that epigenetic subtypes in GBM are more complex than a simple binary model and that a subset of CIMP-negative GBMs with widespread promoter hypermethylation may represent a biologically distinct group with unique molecular regulators and clinical behavior [17,18,19]. Moreover, DNA methylation landscapes in gliomas are shaped by multiple factors, including tumor differentiation state [20] and dynamic epigenetic reprogramming during clonal evolution [21,22].

Although G-CIMP gliomas typically have better outcomes, recent studies indicate considerable heterogeneity within this group [14,23,24]. Methylation profiling has identified G-CIMP–low tumors with reduced methylation and poor survival, and G-CIMP–high tumors with extensive methylation and more favorable outcomes [25]. Some IDH-mutant astrocytomas also progress aggressively despite being G-CIMP–positive [26], indicating that IDH status alone cannot fully predict behavior. Current glioma classifications do not integrate genome-wide methylation patterns beyond *IDH* and *MGMT* [27], and potential prognostic methylation events remain underexplored. To address this, we analyzed gene-specific DNA methylation and expression patterns and evaluated their associations with patient survival. Our integrative approach aimed to uncover epigenetic markers that improve prognostic accuracy beyond IDH-based systems.

## 2. Materials and Methods

### 2.1. Data Acquisition and Sample Classification

#### 2.1.1. Data Acquisition and Preprocessing

DNA methylation and gene expression data, along with associated clinical information, were obtained from The Cancer Genome Atlas (TCGA). The discovery cohort comprised the TCGA GBM dataset (N_Firehose Legacy_ = 285), and the validation cohort used the TCGA GBMLGG dataset (N = 657, including GBM and lower-grade gliomas). Data preprocessing was performed in R (v4.4.3). Methylation levels were represented as β-values (0–1), and RNA-seq data were log_2_-transformed normalized counts. Only samples with complete methylation, expression, and clinical data were retained for analysis.

#### 2.1.2. Classification of Methylation Phenotypes

Tumors were classified using established glioma molecular and epigenetic frameworks as described by Noushmehr et al., Ceccarelli et al., and the TCGA Consortium. Canonical G-CIMP status was defined by IDH mutation status: IDH-mutant diffuse gliomas were classified as G-CIMP, and IDH-wildtype tumors as non-G-CIMP. Because the TCGA-GBM discovery cohort consists almost entirely of IDH-wildtype glioblastomas, no canonical G-CIMP tumors were present in this cohort, and all samples were classified as non-G-CIMP. Canonical G-CIMP stratification was applied only to the independent TCGA-GBMLGG validation cohort, where IDH mutation status and tumor grade information were available. Global DNA methylation levels were evaluated only as descriptive features and were not used for subtype assignment. This IDH-based definition was used to avoid reliance on arbitrary global methylation thresholds and to ensure consistency with established G-CIMP classifications (Supplementary Table S1). This approach captures the canonical, IDH-associated G-CIMP phenotype but does not represent a genome-wide methylation classifier (e.g., the Heidelberg classifier).

### 2.2. Validation of Canonical G-CIMP Methylation Patterns

To confirm that our stratification captured canonical G-CIMP biology, we examined promoter methylation of 10 established G-CIMP marker genes (*PDGFRA*, *EGFR*, *CDKN2A*, *MGMT*, *RBP1*, *GATA6*, *SOX10*, *DCC*, and two *HOXA* family genes) in the TCGA-GBMLGG validation cohort. Promoter CpG β-values were averaged per sample, and group comparisons used Wilcoxon rank-sum tests with Benjamini–Hochberg (BH) correction (adj. *p* < 0.05).

### 2.3. Genome-Wide Methylation Analysis

In the discovery cohort, genome-wide differential methylation between G-CIMP and non-CIMP tumors was assessed using Mann–Whitney U tests at each CpG site, with BH correction across all loci (adj. *p* < 0.05). Mean β-value differences between groups indicated the direction and magnitude of methylation changes. The top 50 differentially methylated CpGs (ranked by adjusted *p*-value) were used for unsupervised hierarchical clustering to visualize subtype separation. In the validation cohort, we focused on validating key differentially methylated genes identified in the discovery cohort, comparing promoter methylation between groups using Wilcoxon tests with BH correction (q < 0.05).

### 2.4. Methylation-Expression Coupling Analysis

#### 2.4.1. Differential Gene Expression Analysis

Differential gene expression analyses were performed to investigate transcriptional differences associated with DNA methylation patterns and to identify candidate epigenetically regulated genes relevant to glioma biology and patient outcome. Analyses focused on two primary categories of genes: (i) core DNA methylation–associated regulators (*DNMT1*, *DNMT3A*, *DNMT3B*, *TET1*, *TET2*, *TET3*, *MECP2*, *MBD1*, *MBD2*, *MBD3*, and *MBD4*), and (ii) genes identified as differentially methylated between tumor subgroups in genome-wide methylation analyses.

In the TCGA-GBM discovery cohort, log_2_-normalized expression values were compared between tumors using unpaired *t*-tests. Because this cohort consists predominantly of IDH-wildtype glioblastomas and does not include canonical G-CIMP tumors, expression analyses were conducted without G-CIMP stratification and used to identify gene-level expression patterns associated with differential methylation status. Nominal statistical significance was defined as *p* < 0.05, and multiple-testing correction was applied using the Benjamini–Hochberg method, with adjusted *p* < 0.05 considered significant.

In the TCGA-GBMLGG validation cohort, differential expression analyses were restricted to candidate genes identified in the discovery phase, including *TUBB2A*, *TM4SF1*, *SCD5*, and selected DNA methylation regulators. Expression levels were compared between canonical G-CIMP (IDH-mutant) and non-G-CIMP (IDH-wildtype) tumors using the same statistical framework. These analyses assessed the reproducibility and generalizability of gene-specific expression differences across a molecularly heterogeneous glioma population encompassing multiple tumor grades. Together, these expression analyses provided a foundation for subsequent integrative assessment of methylation–expression coupling and for prioritizing candidate genes for downstream survival analyses.

#### 2.4.2. Integration of Methylation and Expression Data

To assess the functional relevance of differential DNA methylation, integrative analyses were performed to evaluate the relationship between promoter methylation and gene expression. For genes exhibiting significant promoter methylation differences, corresponding mRNA expression levels were examined across samples to determine whether methylation status was associated with transcriptional regulation. Promoter methylation levels were summarized as mean β-values across promoter-associated CpG probes for each gene and sample. Promoter-associated CpG probes were defined based on Illumina array annotation of gene promoter regions, and all probes mapping to a given promoter were included in the calculation. Spearman rank correlation analyses were then conducted to evaluate the relationship between promoter methylation and log_2_-normalized gene expression levels. Correlations were computed across samples within each cohort, and the strength and direction of association were reported. Genes were classified as exhibiting canonical methylation–expression coupling when increased promoter methylation was associated with reduced gene expression, consistent with transcriptional repression. Genes that did not show a clear inverse relationship were classified as exhibiting non-canonical or context-dependent coupling, reflecting more complex regulatory behavior. Representative scatter plots were generated to visualize these relationships for selected candidate genes, including *TUBB2A* and *TM4SF1*.

These integrative analyses were performed separately in the discovery and validation cohorts to assess the consistency of methylation–expression relationships across datasets with differing molecular composition. Results from these analyses informed the prioritization of candidate genes for downstream survival analyses and interpretation of context-dependent regulatory patterns.

### 2.5. Analysis of DNA Methylation Regulators

Expression and promoter methylation of key methylation regulators (*MECP2*, *MBD1*, *MBD3*, *DNMT3A*) were compared between groups in both cohorts using *t*-tests for expression differences and Wilcoxon tests for methylation differences. These analyses evaluated whether G-CIMP tumors display alterations in epigenetic machinery that establish or maintain their hypermethylated state.

### 2.6. Survival and Prognostic Analysis

#### 2.6.1. Discovery Cohort (TCGA-GBM)

Kaplan–Meier survival analyses evaluated prognostic associations of molecular features in the discovery cohort. Samples were dichotomized by median expression for candidate genes (*TUBB2A*, *TM4SF1*, *CCDC91*, *TGFB2*, *WHSC1*), and survival distributions were compared using log-rank tests.

#### 2.6.2. Validation Cohort (TCGA-GBMLGG)

In the validation cohort, patients were stratified by median expression or methylation of key discovery genes (particularly *TUBB2A*, *TM4SF1*, *SCD5*). Kaplan–Meier curves were generated, and log-rank tests and hazard ratios from univariate Cox models were used to assess the robustness and generalizability of prognostic associations across glioma grades.

#### 2.6.3. Multivariable Survival Analysis

To assess the independent prognostic value of candidate epigenetically regulated genes, multivariable Cox proportional hazards regression models were fitted in the TCGA-GBMLGG validation cohort. Models included age at diagnosis, tumor grade, IDH mutation status, 1p/19q codeletion status, and MGMT promoter methylation status (when available), together with gene-specific promoter methylation levels for *TUBB2A* and *TM4SF1*. Promoter methylation variables were entered into the Cox proportional hazards models as continuous β-values (range 0–1), and hazard ratios were interpreted per unit increase in promoter methylation. Hazard ratios (HRs), 95% confidence intervals (CIs), and Wald *p*-values were reported. Proportional hazards assumptions were evaluated using Schoenfeld residuals.

### 2.7. Statistical Analysis

All statistical tests were two-sided, with *p* < 0.05 considered significant. False discovery rate (FDR) correction using the Benjamini–Hochberg method was applied to multiple comparisons, as indicated. Data visualization (volcano plots, heatmaps, scatter plots, Kaplan–Meier curves) was performed using the R packages ggplot2 and survminer.

## 3. Results

### 3.1. Cohort Characteristics and Canonical G-CIMP Stratification

#### 3.1.1. Discovery Cohort (TCGA-GBM)

The TCGA-GBM discovery cohort consisted predominantly of IDH-wildtype glioblastomas. Because canonical G-CIMP is tightly linked to IDH mutation status, no G-CIMP tumors were present in this cohort. Accordingly, the discovery dataset was used exclusively to identify gene-level DNA methylation–expression relationships and candidate prognostic loci, without applying G-CIMP stratification.

#### 3.1.2. Validation Cohort (TCGA-GBMLGG)

**Figure 1:**
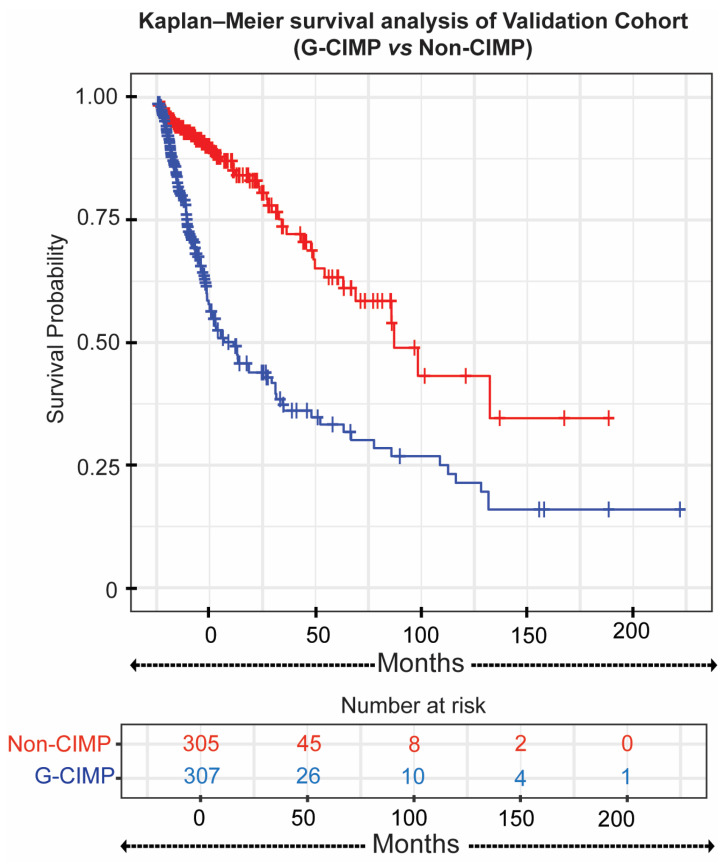
Kaplan–Meier survival analysis using canonical IDH-defined G-CIMP classification in the TCGA-GBMLGG validation cohort. Overall survival is shown for G-CIMP (IDH-mutant) and non-G-CIMP (IDH-wildtype) tumors. G-CIMP tumors exhibited significantly longer overall survival compared with non-G-CIMP tumors (log-rank *p* < 0.0001). Number-at-risk tables are shown below. Blue curves represent G-CIMP (IDH-mutant) tumors and red curves represent non-G-CIMP (IDH-wildtype) tumors.

To evaluate the robustness and clinical relevance of canonical G-CIMP classification beyond GBM, we analyzed the TCGA-GBMLGG cohort (n = 657), which comprises WHO grades II–IV diffuse gliomas and includes a substantial proportion of IDH-mutant tumors. Using canonical IDH-defined G-CIMP classification, the validation cohort demonstrated a significant separation in overall survival between G-CIMP and non-G-CIMP tumors (log-rank *p* < 0.0001; Figure 1). G-CIMP tumors showed significantly longer overall survival compared with non-G-CIMP tumors. This pattern is consistent with the established association between IDH mutation–defined G-CIMP status and favorable clinical outcome.

### 3.2. Validation of Canonical G-CIMP Markers

**Figure 2:**
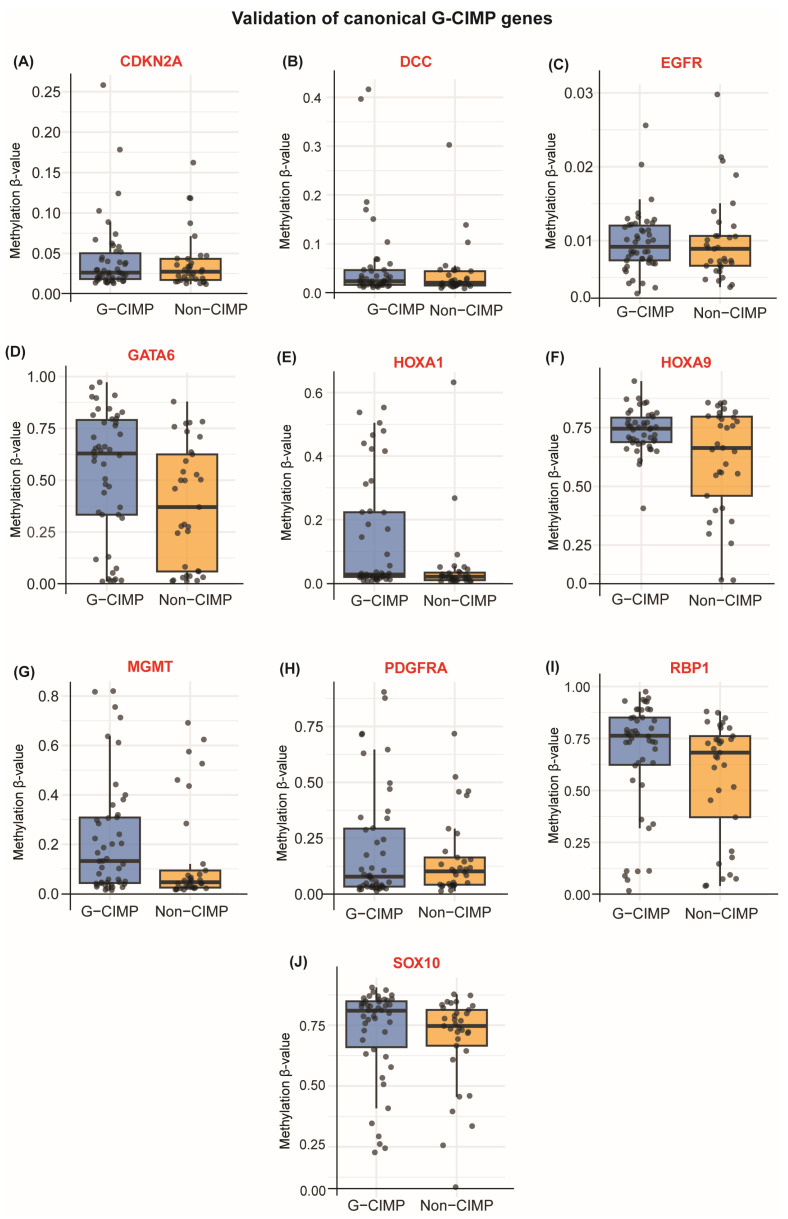
Promoter methylation of established G-CIMP marker genes stratified by IDH status. Boxplots show promoter methylation β-values for canonical G-CIMP marker genes in IDH-mutant (G-CIMP) versus IDH-wildtype (non-G-CIMP) gliomas. Panels (**A**–**J**) correspond to *CDKN2A*, *DCC*, *EGFR*, *GATA6*, *HOXA1*, *HOXA9*, *MGMT*, *PDGFRA*, *RBP1*, and *SOX10*.

To confirm that tumors classified using canonical G-CIMP criteria exhibit expected methylation features, we examined promoter methylation of established G-CIMP marker genes. The distribution of promoter β-values for each marker (Figure 2A–J) showed a clear and consistent shift toward higher methylation in G-CIMP tumors.

Across the TCGA-GBMLGG validation cohort, eight of the ten genes showed higher promoter methylation in G-CIMP tumors, with several reaching nominal significance (Table 1). These results confirm strong subtype-specific methylation enrichment.

Markers showing the most pronounced difference, *RBP1*, *SOX10*, *GATA6*, *HOXA1*, and *HOXA9*, are key regulators of developmental and lineage-specific programs. Their coordinated hypermethylation is a defining feature of the G-CIMP phenotype. Additional canonical markers, including *CDKN2A*, *DCC*, and *PDGFRA*, also showed significantly higher methylation in G-CIMP tumors, though with smaller effect sizes. In contrast, *MGMT* and *EGFR* showed no significant methylation differences between subtypes (adjusted *p* > 0.2). *MGMT* promoter methylation occurs independently of global CIMP status, whereas EGFR activation in GBM is typically driven by gene amplification rather than promoter hypomethylation. Their lack of differential methylation thus reflects expected biology rather than classifier limitations. Collectively, these results are consistent with prior reports describing promoter hypermethylation of established G-CIMP marker genes in IDH-mutant gliomas and support the use of canonical G-CIMP classification for downstream analyses.

### 3.3. Genome-Wide Methylation Differences Between Subtypes

To characterize broader methylation alterations distinguishing G-CIMP from non-CIMP GBMs, we performed an unbiased genome-wide differential methylation analysis. After stringent false discovery rate correction (FDR < 0.05), 11 CpG loci, each mapping to a distinct gene, remained statistically significant (Table 2 and Table S2).

Although moderate in number relative to the >400,000 CpGs assessed, these loci represent the most reproducible subtype-specific methylation differences in the discovery cohort. Among these sites, *MTCH2*, *TUBB2A*, *TGFB2*, and *DACT3* were significantly hypermethylated in G-CIMP tumors, whereas *WHSC1*, *CCDC91*, *STOML3*, *HIPK3*, *NCRNA00095*, *IL27RA*, and *TM4SF1* were hypomethylated. Promoter-level summaries further confirmed that *TM4SF1* exhibited substantially higher methylation in non-CIMP tumors (Δβ ≈ +0.10), despite modest variability among individual probes within the locus (Supplementary Table S2). These findings illustrate that differential methylation in GBM involves both promoter and locus-specific contributions.

**Figure 3:**
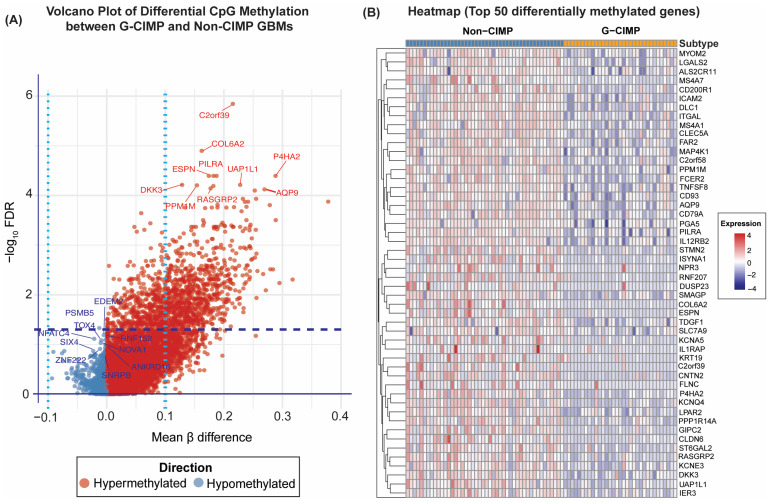
Genome-wide differential methylation analysis in the discovery cohort. (**A**) Volcano plot showing genome-wide CpG methylation differences between G-CIMP and non-CIMP GBMs. Significantly hypermethylated loci are highlighted in red, hypomethylated in blue (FDR < 0.05), and (**B**) Heatmap of the top 50 differentially methylated CpG loci illustrating clear segregation between G-CIMP and non-CIMP tumors based on promoter β-values.

The genome-wide distribution of differential methylation is illustrated in Figure 3A. The volcano plot shows a strong skew toward hypermethylated loci in CIMP-high tumors (red points shifted to the right), along with a smaller but distinct cluster of hypomethylated loci in non-CIMP tumors (blue points moved to the left). Although many CpGs showed nominal group differences, only loci with highly consistent methylation shifts across samples achieved genome-wide significance, reflecting both the biological coherence of the CIMP phenotype and the stringency of multiple-testing correction. Unsupervised clustering of the top 50 differentially methylated CpGs (ranked by adjusted *p*-value) provided further confirmation of subtype separation. As shown in Figure 3B, G-CIMP tumors displayed uniformly elevated β-values across these CpGs, whereas non-CIMP tumors were consistently hypomethylated. The clear segregation of samples indicates coordinated methylation differences across these loci rather than isolated gene-level events.

Notably, well-established tumor suppressor genes such as RB1, *CDKN2A*, and *PTEN* did not appear among the CpGs that were significantly discriminative. This is expected: these loci are often methylated in both G-CIMP and non-CIMP tumors, diminishing their utility as subtype-defining markers. Their absence reinforces prior observations that GBM harbors widespread promoter methylation independent of CIMP status. Overall, these genome-wide findings demonstrate that although only a few CpGs meet stringent statistical thresholds, the directionality, consistency, and clustering behavior of these loci support a robust epigenetic distinction between tumors stratified by differential methylation patterns in the TCGA-GBM discovery cohort. These signatures form a strong quantitative foundation for downstream integrative analyses of transcriptional regulation, pathway involvement, and clinical implications.

### 3.4. Methylation–Expression Coupling of Key Genes

**Figure 4:**
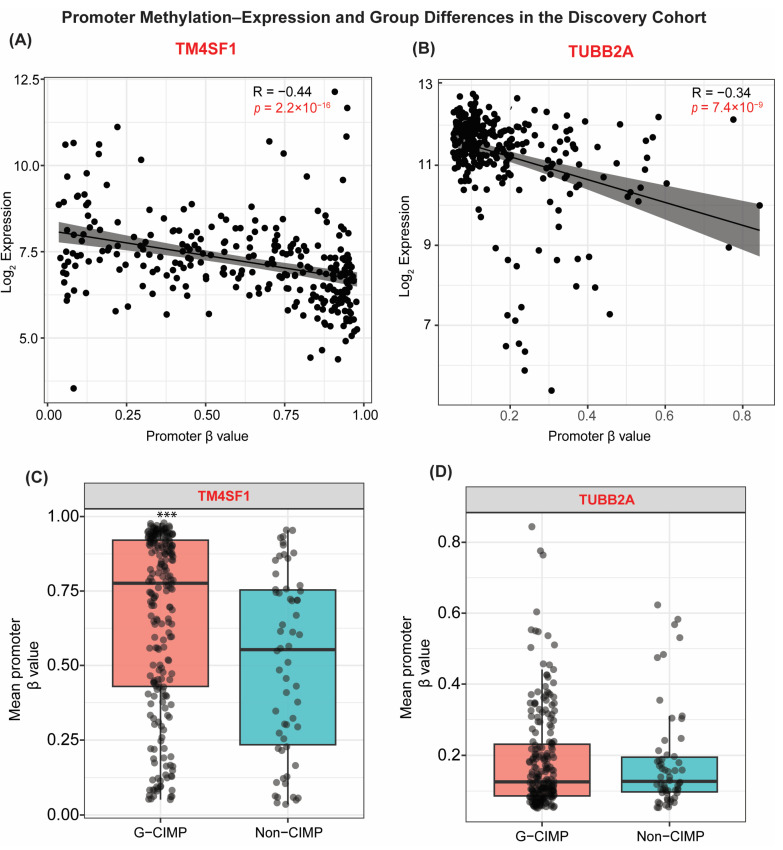
Promoter methylation–expression coupling and group-wise methylation differences for *TM4SF1* and *TUBB2A* in the TCGA-GBM discovery cohort. (**A**) Scatterplot showing the relationship between TM4SF1 promoter methylation (β-value) and mRNA expression (log_2_-transformed) across GBM samples. TM4SF1 demonstrates a modest inverse monotonic association between promoter methylation and transcript abundance (Spearman ρ = −0.44, *p* = 2.2 × 10^−16^). (**B**) Scatterplot showing promoter methylation–expression coupling for *TUBB2A*. Higher promoter methylation is associated with reduced mRNA expression (Spearman ρ = −0.34, *p* = 7.4 × 10^−9^). (**C**,**D**) Group-wise comparison of promoter methylation (mean β-value) between G-CIMP and non-CIMP tumors for (**C**) *TM4SF1* and (**D**) *TUBB2A*. Boxes represent the interquartile range with the median line; whiskers correspond to the standard 1.5 × IQR convention. Points represent individual tumor samples. Statistical significance was annotated as follows: *** adjusted *p* < 0.001 (Benjamini–Hochberg correction).

To determine whether promoter methylation differences translated into functional transcriptional changes, we examined mRNA expression for the genes identified as significantly differentially methylated in the discovery cohort. Five representative loci, *TUBB2A*, *CCDC91*, *TGFB2*, *WHSC1*, and *TM4SF1*, were selected to capture both hypermethylated and hypomethylated patterns. In the TCGA-GBM discovery cohort, four genes (*TUBB2A*, *CCDC91*, *TGFB2*, and *WHSC1*) displayed expression profiles consistent with canonical epigenetic repression: each showed significantly lower mRNA levels in G-CIMP tumors (*p* < 0.01), mirroring their higher promoter methylation. These relationships indicate that promoter hypermethylation is associated with transcriptional downregulation in the hypermethylated subtype. Conversely, *TM4SF1* exhibited higher expression in G-CIMP tumors, reflecting the relative hypomethylation observed at this locus (Figure 4A–D). *TM4SF1* nonetheless demonstrated an inverse methylation–expression correlation, indicating a functional relationship between methylation and transcription. Still, its regulatory behavior differed from that of other genes, suggesting that additional context-dependent mechanisms beyond promoter methylation alone may govern *TM4SF1*.

To further evaluate whether these associations were influenced by extreme values, sensitivity analyses were performed using Kendall’s rank correlation and 1% winsorized data. For *TUBB2A*, the inverse association remained stable (initial Spearman ρ = −0.34; trimmed ρ = −0.32; Kendall τ = −0.23). For *TM4SF1*, the association was similarly robust (initial ρ = −0.44; trimmed ρ = −0.45; Kendall τ = −0.31). These findings confirm that the reported correlations are not driven by a small subset of extreme observations (Supplementary Table S3).

**Figure 5:**
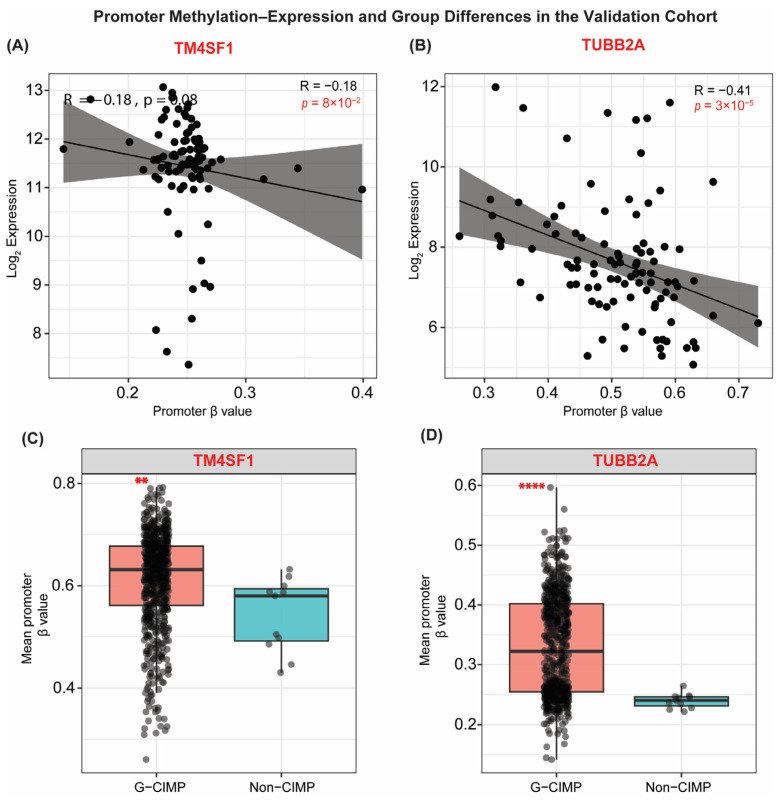
Promoter methylation–expression coupling and group-wise promoter methylation differences for *TM4SF1* and *TUBB2A* in the TCGA-GBMLGG validation cohort. (**A**) Scatterplot showing the relationship between *TM4SF1* promoter methylation (β-value) and log_2_-transformed mRNA expression across validation cohort samples. *TM4SF1* shows a weak, non-significant inverse correlation (Spearman R = −0.18, *p* = 0.08). (**B**) Scatterplot illustrating promoter methylation–expression coupling for *TUBB2A*. Higher promoter methylation is significantly associated with reduced expression (Spearman R = −0.41, *p* = 3 × 10^−5^). (**C**) Boxplot comparing mean promoter β-values for *TM4SF1* between G-CIMP and non-CIMP tumors. *TM4SF1* shows significantly higher promoter methylation in G-CIMP tumors (*p* < 0.01), and (**D**) Boxplot comparing mean promoter β-values for TUBB2A between G-CIMP and non-CIMP tumors. *TUBB2A* demonstrates markedly higher promoter methylation in G-CIMP tumors (**** *p* < 0.0001). Each point represents a single tumor sample; boxes denote the interquartile range with a median line. Statistical significance was annotated as follows: ** adjusted *p* < 0.01; **** adjusted *p* < 0.0001 (Benjamini–Hochberg correction).

We next evaluated whether these relationships persisted in the TCGA-GBMLGG validation cohort. *TUBB2A* again demonstrated strong inverse coupling, with G-CIMP tumors showing pronounced promoter hypermethylation accompanied by significantly reduced expression (adj. *p* = 3.1 × 10^−25^), supporting *TUBB2A* as a robust gene whose expression is consistently associated with promoter methylation across cohorts. In contrast, TM4SF1 showed lower expression in G-CIMP tumors in the validation cohort, consistent with the higher promoter methylation observed in this dataset. These cohort-dependent differences indicate that TM4SF1 methylation–expression associations are influenced by the underlying molecular composition, including IDH mutation status and tumor grade, rather than reflecting an actual biological reversal of regulatory direction (Figure 5A–D).

Other differentially methylated genes, including *WHSC1* and *CCDC91*, showed directionally consistent repression in G-CIMP tumors in the validation cohort. However, effect sizes were modest, reflecting the greater biological heterogeneity of the mixed-grade GBMLGG dataset. Finally, cross-cohort analysis revealed that *TUBB2A* and *TM4SF1* both exhibited clear inverse methylation–expression relationships, confirming functional coupling at these loci. By contrast, *MECP2*, despite being significantly upregulated in G-CIMP tumors, showed neither promoter methylation differences nor methylation–expression correlation, illustrating that not all transcriptional alterations in G-CIMP tumors arise from promoter methylation mechanisms (Supplementary Table S4).

Overall, these findings indicate that G-CIMP tumors exhibit a coordinated epigenetic–transcriptional program in which promoter hypermethylation contributes to gene silencing at a key subset of loci, most notably *TUBB2A*. In contrast, other genes, such as *TM4SF1*, exhibit more complex, context-dependent regulatory behavior across glioma subtypes. To assess whether cohort-dependent differences reflected underlying molecular heterogeneity, we examined methylation–expression relationships within homogeneous molecular strata. When analyses were restricted to IDH-mutant tumors, *TM4SF1* promoter methylation retained an inverse association with gene expression, although effect sizes were attenuated (Supplementary Table S5). In IDH-wildtype tumors, the association was weaker and more variable. Similar patterns were observed when analyses were stratified by tumor grade. These findings indicate that apparent cohort-dependent differences are primarily due to differences in IDH status and grade distribution rather than to an actual biological inversion.

### 3.5. Differential Expression of DNA Methylation Regulators

To determine whether G-CIMP tumors harbor alterations in epigenetic machinery that help establish or maintain their hypermethylated state, we evaluated the expression of key DNA methylation regulators in the discovery cohort. Several regulators showed significantly higher expression in G-CIMP compared with non-CIMP tumors, including *MECP2*, *MBD3*, *DNMT3A*, and *MBD1* (each adj. *p* < 0.01; Table 3). These genes encode essential components of DNA methylation control: *DNMT3A* mediates de novo methylation, while *MECP2*, *MBD1*, and *MBD3* bind methylated CpG sites and help recruit chromatin-remodeling complexes such as the NuRD complex.

The coordinated upregulation of these regulators suggests that G-CIMP tumors may reinforce their hypermethylated state by increasing their methylation “writing” and “reading” capacity. This pattern indicates that the G-CIMP phenotype is not solely the downstream consequence of IDH mutation but may involve active stabilization of methylation-dependent transcriptional repression. Other regulators, including *DNMT1*, *DNMT3B*, *MBD2*, and *TET1–3*, did not differ between groups, indicating that only specific components of the DNA methylation apparatus participate in G-CIMP–associated regulatory remodeling.

In the validation cohort, methylation regulator expression patterns were more heterogeneous, consistent with the broader spectrum of glioma grades and IDH statuses represented. Among the regulators elevated in the discovery cohort, only *MECP2* remained significantly upregulated in G-CIMP tumors (adj. *p* = 0.0038). *MBD1* showed a nonsignificant trend toward increased expression, while *MBD3* and *DNMT3A* did not display subtype-specific differences. These discrepancies suggest that some regulatory changes observed in GBM may not generalize uniformly across lower-grade IDH-mutant gliomas and may instead reflect grade-dependent or lineage-specific epigenetic requirements.

Notably, *MECP2* overexpression occurred despite no differences in promoter methylation, indicating that its regulation is independent of the promoter-level CpG state. This finding suggests that *MECP2* may be selectively upregulated in hypermethylated tumors to help maintain transcriptional repression across a broad set of methylated loci. Together, these data show that G-CIMP tumors exhibit a selective, partially conserved epigenetic regulatory profile, characterized by the enhancement of specific methylation-writing and methylation-interpreting genes. This regulatory environment likely contributes to the stability and functional impact of the G-CIMP epigenetic program.

### 3.6. Prognostic Significance of Epigenetically Regulated Genes

To determine whether epigenetically regulated genes contribute to clinical heterogeneity within the G-CIMP subtype, we assessed the prognostic relevance of expression and promoter methylation for the top differentially methylated candidates identified in earlier analyses.

#### 3.6.1. Discovery Cohort (TCGA-GBM)

**Figure 6:**
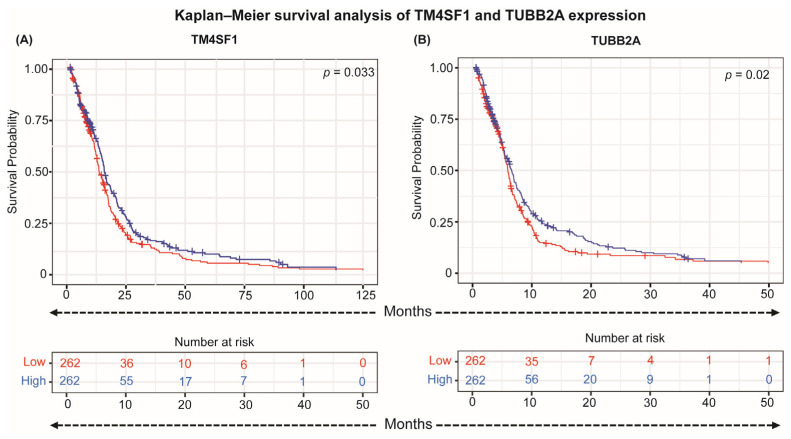
Prognostic significance of candidate gene expression in the discovery cohort. Kaplan–Meier survival analyses for TCGA-GBM discovery cohort. (**A**) *TM4SF1* expression (borderline association; log-rank *p* = 0.033) and (**B**) *TUBB2A* expression (significant association; log-rank *p* = 0.02). Patients were dichotomized into high- and low-expression groups based on the median expression value. Blue curves represent the high-expression group and red curves represent the low-expression group. High expression of either gene was associated with shorter overall survival.

In the TCGA-GBM discovery cohort, *TUBB2A* expression was significantly associated with overall survival. Patients with high *TUBB2A* expression experienced notably shorter survival than those with low expression (log-rank *p* = 0.02; Figure 6B). This finding suggests that incomplete silencing of *TUBB2A* may contribute to more aggressive tumor behavior or reduced therapeutic responsiveness.

By contrast, *TM4SF1* expression exhibited only a borderline association with survival (log-rank *p* = 0.033; Figure 6A). Although higher expression tended to correlate with poorer outcomes, the association did not reach statistical significance, and other differentially methylated genes (e.g., *TGFB2*, *CCDC91*, *WHSC1*) showed no survival differences. These results indicate that not all epigenetically altered genes have prognostic impact within the TCGA-GBM cohort.

#### 3.6.2. Validation Cohort (TCGA-GBMLGG)

We next evaluated whether prognostic associations observed in GBM extended to the broader, more clinically heterogeneous TCGA-GBMLGG cohort. For *TUBB2A*, the validation dataset confirmed a strong predictive effect. Still, at the level of methylation rather than expression, tumors with high promoter methylation corresponding to transcriptional repression had significantly improved overall survival (log-rank *p* < 2 × 10^−16^; HR ≈ 0.15). This is entirely consistent with findings from the discovery cohort, which showed that high *TUBB2A* activity was associated with adverse outcomes, reinforcing its role as a potential epigenetically regulated driver of tumor aggressiveness.

For *TM4SF1*, both gene expression and promoter methylation were significantly associated with survival in the validation cohort (*p* < 0.0001 for each). Tumors with high *TM4SF1* expression or low promoter methylation exhibited worse prognosis, whereas tumors with *TM4SF1* promoter hypermethylation and corresponding transcriptional suppression showed improved survival. These results suggest that *TM4SF1* may function as a context-dependent oncogenic factor, promoting a more aggressive or treatment-resistant disease phenotype.

Together, survival findings across both cohorts highlight *TUBB2A* and *TM4SF1* as reproducible prognostic markers modulated by epigenetic state. *TUBB2A* appears detrimental when expressed, supporting the beneficial impact of promoter hypermethylation and transcriptional silencing. *TM4SF1* demonstrates cohort-specific regulatory behavior yet consistently associates with poorer outcomes when active. These results underscore that, even within the clinically favorable G-CIMP subgroup, epigenetic heterogeneity shapes patient survival, revealing biologically meaningful substructure within the G-CIMP classification.

#### 3.6.3. Multivariable Survival Analysis

To assess whether *TUBB2A* and *TM4SF1* provide prognostic information beyond established clinical and molecular factors, multivariable Cox proportional hazards regression analyses were performed in the TCGA-GBMLGG validation cohort. Models included age at diagnosis, tumor grade, IDH mutation status, 1p/19q co-deletion status, and MGMT promoter methylation status, together with gene-specific promoter methylation levels for *TUBB2A* and *TM4SF1*. Promoter methylation variables were entered into the Cox proportional hazards models as continuous β-values (range 0–1), and hazard ratios were interpreted per unit increase in promoter methylation.

As expected, increasing age at diagnosis and higher tumor grade were independently associated with poorer overall survival. After multivariable adjustment, promoter methylation of *TUBB2A* demonstrated a strong protective effect (HR = 0.017, 95% CI: 0.0003–1.06) with borderline statistical significance (*p* = 0.053), indicating that its prognostic association is partly independent of established clinical and molecular determinants. In contrast, the association between *TM4SF1* promoter methylation and survival was attenuated after adjustment and did not remain independently significant (*p =* 0.59).

These findings indicate that, while both genes show prognostic associations in univariate analyses, *TUBB2A* exhibits a partially independent relationship with outcome, whereas *TM4SF1* appears more context-dependent and influenced by underlying tumor characteristics. As shown in Table 4.

## 4. Discussion 

Our study shows that IDH-defined G-CIMP gliomas are not a single molecular state but exhibit heterogeneity in regulatory and prognostic features. Using two large TCGA cohorts [28,29], we identify *TUBB2A* and *TM4SF1* as consistent, methylation-regulated prognostic markers across IDH backgrounds and tumor grades. G-CIMP tumors are associated with increased expression of methylation-associated regulators, such as *MECP2* and *DNMT3A*, suggesting a potential role for these factors in maintaining a hypermethylated state. Because G-CIMP status was assigned based on IDH mutation status, this framework reflects canonical G-CIMP biology but does not constitute a genome-wide methylation-based tumor classification.

In addition to canonical IDH-driven G-CIMP stratification, our results indicate substantial heterogeneity within G-CIMP tumors that is captured by gene-specific methylation and transcriptional regulation [19,20]. Gene-specific methylation effects vary by context, as seen in differences in TM4SF1 between IDH-wild-type and IDH-mutant gliomas and in *MECP2* expression uncoupled from promoter methylation. Together, these findings indicate that G-CIMP classification obscures biologically meaningful subgroups identified by gene-specific methylation and transcriptional regulation.

The foundational analyses by Noushmehr et al. and Ceccarelli et al. defined G-CIMP as an IDH-driven hypermethylation state linked to better prognosis [12,14]. Our study extends this by identifying functional modules within G-CIMP, including variation in methylation-maintenance genes (*MECP2*, *DNMT3A*) and new methylation-regulated prognostic genes (*TUBB2A*, *TM4SF1*). Our analyses extend prior work by highlighting heterogeneity within G-CIMP tumors driven by differential regulation of methylation-maintenance machinery and gene-specific methylation events [14]. Thus, while earlier studies treated G-CIMP as relatively uniform, our data show clear subclass structure.

Earlier classifications based on global methylation levels identified survival differences but provided limited mechanistic resolution [25,30]. Our work advances this by identifying specific CpG sites and genes that distinguish subgroups and linking them to gene expression and survival. We also observe that methylation–expression relationships differ across cohorts, particularly for *TM4SF1* and *SCD5*, consistent with context-dependent regulation. Gene-specific methylation patterns reveal heterogeneity within G-CIMP tumors that is not captured by broad methylation-based classifications alone. While prior studies have emphasized global methylation, we identify the key regulatory genes that drive variation within G-CIMP.

Previous studies have demonstrated that DNA methylation patterns are strongly influenced by IDH mutation status and tumor differentiation state in diffuse gliomas [12,13,14,15,16,20]. Our findings suggest that context-dependent methylation at specific loci, such as *SCD5* and *TM4SF1*, may reflect differences in epigenetic state associated with IDH mutation status and tumor grade. These context-dependent effects may help explain previously reported inconsistencies in prognostic associations across glioma subtypes [14,16,21,22,23,24,25]. This work improves risk stratification within IDH-defined G-CIMP gliomas by identifying gene-specific methylation events associated with patient outcome [12,13,14,25,29,30,31,32,33]. *TUBB2A* and *TM4SF1* provide additional prognostic information beyond IDH status alone, although their biological roles remain incompletely elucidated. *TUBB2A* encodes a β-tubulin isoform that contributes to α/β-tubulin heterodimer formation and microtubule assembly. Microtubules are essential for mitotic spindle organization, intracellular trafficking, and cytoskeletal remodeling, processes directly linked to cellular proliferation and tumor aggressiveness [34,35]. Alterations in tubulin dynamics have been implicated in multiple malignancies and are known to influence tumor cell division and migration [35,36]. The association between *TUBB2A* promoter methylation, transcriptional repression, and improved survival, therefore, suggests a biologically plausible link between epigenetic regulation and proliferative capacity.

*TM4SF1* (Transmembrane 4 L Six Family Member 1) is a tetraspanin-like membrane protein involved in membrane microdomain organization and regulation of cell adhesion and motility. *TM4SF1* has been implicated in tumor invasion, angiogenic signaling, and tumor–microenvironment interactions across several solid tumors [37,38,39]. In glioma, membrane-associated signaling pathways and cytoskeletal remodeling contribute to invasive growth patterns [32,39]. The observed relationship between TM4SF1 methylation, expression, and clinical outcome is therefore consistent with a potential role in modulating tumor aggressiveness.

Altered expression of methylation-associated regulators, such as MECP2 and DNMT3A, is consistent with prior reports of epigenetic remodeling in G-CIMP tumors; however, functional studies will be required to elucidate causal mechanisms and therapeutic relevance.

Several limitations warrant consideration. In addition, G-CIMP status was assigned using an IDH-based definition and therefore reflects canonical G-CIMP biology rather than the full spectrum of methylation-defined glioma subtypes identified by genome-wide classifiers [31,32]. Second, although the associations were strong, we did not test whether *TUBB2A*, *TM4SF1*, *SCD5*, or *MECP2* directly drives these effects, and experimental validation is required [33]. Third, the survival findings are based on retrospective TCGA data with heterogeneous treatment regimens, which may influence the observed prognostic associations. Fourth, TCGA primarily represents Western populations, and validation in more diverse cohorts is essential.

## 5. Conclusions

This study refines the understanding of the glioma CpG island methylator phenotype (G-CIMP) by demonstrating substantial molecular and prognostic heterogeneity within IDH-defined G-CIMP tumors. Through integrative analyses of DNA methylation and gene expression of two large TCGA cohorts, we identify *TUBB2A* and *TM4SF1* as gene-specific loci whose promoter methylation is associated with patient outcome. Multivariable survival analyses incorporating established clinical and molecular determinants indicate that *TUBB2A* promoter methylation exhibits a strong protective effect, partially independent of age, tumor grade, IDH mutation status, 1p/19q co-deletion, and MGMT promoter methylation. In contrast, the prognostic association of *TM4SF1* appears more context-dependent. These findings highlight the importance of gene-specific epigenetic regulation in shaping glioma behavior beyond broad molecular classifications. Overall, our results underscore the value of integrating targeted methylation markers with canonical molecular features to improve prognostic stratification in diffuse glioma and provide a focused framework for future functional and clinical validation.

## Figures and Tables

**Table 1:** Promoter methylation of established G-CIMP marker genes in IDH-mutant versus IDH-wildtype gliomas.

Gene Symbol	Mean G-CIMP	Mean Non-CIMP	*p* Value	Adj. *p* Value
*CDKN2A*	0.043	0.039	6.82 × 10^−1^	6.82 × 10^−1^
*DCC*	0.054	0.039	4.09 × 10^−1^	5.84 × 10^−1^
*EGFR*	0.01	0.01	5.11 × 10^−1^	6.38 × 10^−1^
*GATA6*	0.532	0.376	2.02 × 10^−2^	6.95 × 10^−2^
*HOXA1*	0.137	0.049	1.22 × 10^−2^	6.95 × 10^−2^
*HOXA9*	0.738	0.614	5.67 × 10^−2^	1.13 × 10^−1^
*MGMT*	0.221	0.143	2.25 × 10^−2^	6.95 × 10^−2^
*PDGFRA*	0.203	0.16	6.46 × 10^−1^	6.82 × 10^−1^
*RBP1*	0.67	0.56	2.78 × 10^−2^	6.95 × 10^−2^
*SOX10*	0.723	0.69	1.04 × 10^−1^	1.74 × 10^−1^

**Table 2:** Differentially methylated genes between G-CIMP and non-CIMP GBMs in the discovery cohort (key loci).

Gene Symbol	Mean G-CIMP	Mean Non-CIMP	Mean Difference	*p* Value	Adj. *p* Value	Direction
*TM4SF1*	0.658	0.506	0.152	1.00 × 10^−3^	0.0034	Hypermethylated in G-CIMP
*WHSC1*	0.692	0.628	0.063	8.90 × 10^−3^	0.0221	
*IL27RA*	0.118	0.065	0.053	1.16 × 10^−2^	0.0274	
*TGFB2*	0.226	0.165	0.061	1.20 × 10^−2^	0.028	
*SCD5*	0.023	0.021	0.001	1.56 × 10^−1^	0.2206	
*CCDC91*	0.708	0.685	0.023	3.41 × 10^−1^	0.4149	
*TUBB2A*	0.184	0.182	0.002	9.30 × 10^−1^	0.9463	
*MTCH2*	0.135	0.133	0.002	9.42 × 10^−1^	0.9551	
*DACT3*	0.263	0.318	−0.055	9.68 × 10^−2^	0.1512	Hypomethylated in G-CIMP

**Table 3:** Differential expression of candidate genes and DNA methylation regulators between G-CIMP and non-CIMP GBMs in the discovery cohort.

Gene Symbol	*p*-Adj. Value	Differentially Expressed (DE) Status
*MECP2*	1.84 × 10^−5^	Upregulated
*MBD3*	8.78 × 10^−5^	Upregulated
*DNMT3A*	0.001065	Upregulated
*MBD1*	0.006156	Upregulated

**Table 4:** Multivariable Cox proportional hazards regression analysis of overall survival in the TCGA-GBMLGG validation cohort.

Variable	Hazard Ratio (HR)	95% CI	*p*-Value
Age at diagnosis (per year)	1.05	1.04–1.07	2.3 × 10^−11^
Grade G3 vs. reference	1.96	1.18–3.25	0.009
Grade G4 vs. reference	2.58	1.38–4.85	0.003
IDH-wildtype vs. mutant	1.33	0.66–2.67	0.43
1p/19q non-codeleted vs. codeleted	1.72	0.93–3.18	0.084
MGMT promoter unmethylated vs. methylated	1.11	0.74–1.65	0.61
TUBB2A promoter methylation	0.017	0.0003–1.06	0.053
TM4SF1 promoter methylation	0.54	0.06–4.89	0.59

## Data Availability

The datasets analyzed during the current study are publicly available as follows: TCGA GBM (Firehose Legacy): https://www.cbioportal.org/study/summary?id=gbm_tcga (accessed on 6 October 2025); TCGA GBMLGG (Ceccarelli et al., 2016) [14]: https://doi.org/10.1016/j.cell.2015.12.028 (accessed on 6 October 2025).

## Supplementary Materials

The following supporting information can be downloaded at: https://www.mdpi.com/article/10.3390/cancers18040638/s1, Table S1: Sample-level subtype classification for the TCGA-GBM discovery cohort (G-CIMP vs. non-CIMP); Table S2: Complete list of differentially methylated CpG loci (G-CIMP vs. non-CIMP) with *p*-values and FDR adjustments; Table S3: Genome-wide methylation–expression correlation analysis results (Spearman ρ and adjusted *p*); Table S4: Stratified methylation–expression associations by IDH status and tumor grade in the validation cohort; Table S5: Summary of canonical and non-canonical methylation–expression coupling across candidate genes.

## Abbreviations

The following abbreviations are used in this manuscript:

95% CI	95% confidence interval
BAI1	Brain-specific angiogenesis inhibitor 1
BH	Benjamini–Hochberg (false-discovery rate correction)
β-value (β)	Fractional DNA methylation level (0–1) at a CpG site
C2orf39	Chromosome 2 open reading frame 39
CCDC91	Coiled-Coil Domain Containing 91
CGI	CpG island
CI	Confidence interval
CIMP	CpG island methylator phenotype
CNS	Central nervous system
Cox (Cox PH)	Cox proportional hazards regression
CpG	Cytosine–phosphate–guanine dinucleotide
DACT3	Disheveled-binding antagonist of β-catenin 3
DCC	Deleted in Colorectal Carcinoma
Δβ (delta-β)	Difference in mean β-values between groups
DNMT	DNA methyltransferase (DNMT1, DNMT3A, DNMT3B)
DNMTi	DNA methyltransferase inhibitor (e.g., 5-azacytidine)
DNA	Deoxyribonucleic acid
EGFR	Epidermal growth factor receptor
FDR (q)	False discovery rate (adjusted *p*-value)
G-CIMP	Glioma CpG island methylator phenotype
GBM	Glioblastoma
GBMLGG	TCGA combined cohort of GBM and lower-grade gliomas
HDACi	Histone deacetylase inhibitor
HIPK3	Homeodomain Interacting Protein Kinase 3
HOXA1	Homeobox A1
HOXA9	Homeobox A9
HR	Hazard ratio
IDH	Isocitrate dehydrogenase (IDH1/IDH2)
IL27RA	Interleukin 27 receptor subunit alpha
KM	Kaplan–Meier (survival analysis)
logFC	Log2 fold change
Log-rank	Mantel–Cox test for survival curve comparison.
MBD	Methyl-CpG binding domain protein (MBD1, MBD2, MBD3, MBD4)
MECP2	Methyl-CpG binding protein 2
MGMT	O6-methylguanine-DNA methyltransferase
MRPL38	Mitochondrial Ribosomal Protein L38
mRNA	Messenger RNA
MTCH2	Mitochondrial Carrier 2
MTSS1	Metastasis suppressor 1
NCRNA00095	Long non-coding RNA 95
NuRD	Nucleosome remodeling and deacetylase complex
OS	Overall survival
PCA	Principal Component Analysis
PC1	Principal Component 1
PC2	Principal Component 2
PILRA	Paired immunoglobulin-like type 2 receptor alpha.
PTEN	Phosphatase and Tensin Homolog
RB1	Retinoblastoma 1
RBP1	Retinol-binding protein 1
RNA	Ribonucleic acid
RSEM	RNA-Seq by Expectation-Maximization (expression quantification)
RTK2	Receptor tyrosine kinase II (classical/RTK2 GBM subtype; TCGA)
SCD5	Stearoyl-CoA desaturase 5
SDC2	Syndecan 2
SOX10	SRY-box transcription factor 10
Spearman ρ	Spearman’s rank correlation coefficient
SSRP1	Structure-specific recognition protein 1
STAT1	Signal transducer and activator of transcription 1
STOML3	Stomatin-like protein 3
TBC1D23	TBC1 domain family member 23
TCGA	The Cancer Genome Atlas
TGFB2	Transforming growth factor beta 2
TM4SF1	Transmembrane 4 L Six Family Member 1
TUBB2A	Tubulin beta 2A
TUBB3	Tubulin beta 3
TUBB6	Tubulin beta 6
USP10	Ubiquitin-specific peptidase 10
WHSC1	Wolf–Hirschhorn Syndrome Candidate 1
ZNF219	Zinc finger protein 21
